# Serological, Molecular and Entomological Surveillance Demonstrates Widespread Circulation of West Nile Virus in Turkey

**DOI:** 10.1371/journal.pntd.0003028

**Published:** 2014-07-24

**Authors:** Koray Ergunay, Filiz Gunay, Ozge Erisoz Kasap, Kerem Oter, Sepandar Gargari, Taner Karaoglu, Seda Tezcan, Mehmet Cabalar, Yakup Yildirim, Gürol Emekdas, Bulent Alten, Aykut Ozkul

**Affiliations:** 1 Faculty of Medicine, Department of Medical Microbiology, Virology Unit, Hacettepe University, Ankara, Turkey; 2 Faculty of Sciences, Department of Biology, Division of Ecology, Hacettepe University, Ankara, Turkey; 3 Faculty of Veterinary Medicine, Department of Parasitology, Istanbul University, Istanbul, Turkey; 4 Faculty of Veterinary Medicine, Department of Virology, Ankara University, Ankara, Turkey; 5 Faculty of Medicine, Department of Medical Microbiology, Mersin University, Mersin, Turkey; 6 Faculty of Veterinary Medicine, Department of Virology, Harran University, Ankara, Turkey; 7 Faculty of Veterinary Medicine, Department of Virology, Kafkas University, Ankara, Turkey; Aix Marseille University, Institute of Research for Development, and EHESP School of Public Health, France

## Abstract

West Nile virus (WNV), a mosquito-borne flavivirus with significant impact on human and animal health, has recently demonstrated an expanded zone of activity globally. The aim of this study is to investigate the frequency and distribution of WNV infections in potential vectors and several mammal and avian species in Turkey, where previous data indicate viral circulation. The study was conducted in 15 provinces across Turkey during 2011–2013. In addition, the entomological study was extended to 4 districts of the Turkish Republic of Northern Cyprus. WNV exposure was determined in humans, horses, sheep and ducks from Mersin, Sanliurfa, Van and Kars provinces of Turkey, via the detection of neutralizing antibodies. WNV RNA was sought in human and equine samples from Mersin, Adana and Mugla provinces. Field-collected mosquitoes from 92 sites at 46 locations were characterized morphologically and evaluated for viral RNA. Neutralizing antibodies were identified in 10.5% of the 1180 samples studied and detected in all species evaluated. Viral nucleic acids were observed in 5.9% of 522 samples but only in horses. A total of 2642 mosquito specimens belonging to 15 species were captured, where *Ochlerotatus caspius* (52.4%), *Culex pipiens* sensu lato (24.2%) comprise the most frequent species. WNV RNA was detected in 4 mosquito pools (1.9%), that comprise *Oc. caspius Cx. pipiens* s.l. and DNA barcoding revealed the presence of *Cx. quinquefasciatus and Cx. perexiguus* mosquitoes in infected *Culex* pools. All WNV partial sequences were characterized as lineage 1 clade 1a. These findings indicate a widespread WNV activity in Turkey, in Eastern Thrace and Mediterranean-Aegean regions as well as Southeastern and Northeastern Anatolia.

## Author Summary

West Nile virus (WNV), frequently transmitted to humans and other susceptible species via bites of infected mosquitoes, is a significant global public health threat. Limited information is available on WNV epidemiology in Turkey, located in the endemic zone of the agent around the Mediterranean Sea. This study was performed to reveal WNV activity in potential hosts and vector mosquitoes, involving 11 provinces in Turkey and the Turkish Republic of Northern Cyprus during 2011–2013. Our findings indicate virus exposure in humans and various animals in previously unexplored regions as well as a high rate of virus circulation in equine blood samples during the mosquito season. Field-captured mosquito specimens demonstrated the presence of major WNV vectors among 15 species identified. WNV infection was detected in 1.9% of the pooled mosquito specimens. Molecular characterization of the individual mosquitoes of the infected pools revealed *Culex quinquefasciatus* and *Cx. perexiguus* species, important WNV vectors where *Culex quinquefasciatus* not previously known to exist in Turkey. Partial viral genome sequences obtained from infected hosts were characterized as lineage 1, the predominant pathogenic WNV strain circulating in Europe as well as the American Continent.

## Introduction

West Nile virus (WNV) is a re-emerging arthropod-borne virus with a significant impact on human and animal health [1]. WNV demonstrates an extensive zone of distribution throughout Africa, the Middle East, southern Europe, western Russia, southwestern Asia, and Australia [2], [3]. The global epidemiology of WNV has drastically changed during the last decades, with the introduction and spread of the virus in the American continent and increased reporting of virus activity in Europe, probably influenced by the interaction of factors such as global warming, demographic changes and modern transportation [3]–[5]. Since 1990s, the human disease incidence of WNV strains with probable African origin have increased in parts of Russia and southern, central and eastern Europe, with large outbreaks of notable severity occurring in Romania, Russia, Israel, Italy and Greece [3]. In the western hemisphere, WNV has spread from its initial site of detection in 1999 across North America and now circulates in Mexico, South America, and the Caribbean [6], [7]. In the United States, WNV resurgence was observed in 2012 after several years of decreasing incidence [5].

Taxonomically, WNV is classified in the Japanese encephalitis serocomplex of the *Flavivirus* genus within *Flaviviridae* family, along with more than 70 viruses including important human pathogens such as dengue and yellow fever viruses [8], [9]. Similar to other flaviviruses, WNV is an enveloped virus with a single-stranded, positive sense, 11-kb RNA genome that transcribes a single polyprotein, cleaved by host and viral proteases into structural and nonstructural viral proteins [10]. WNV strains can be designated into at least 5 phylogenetic lineages, however, only lineage 1 and 2 isolates have been associated with significant outbreaks in humans [3], [11]. The virus is normally maintained and spread through a bird-mosquito cycle involving ornithophilic mosquitoes belonging to *Culex* species. However, it can also be spread to a wide range of incidental hosts including humans and horses, via mammophilic and/or anthropophilic mosquito species (including *Aedes* spp. and *Ochlerotatus* spp.). WNV has the potential to cause severe illness characterized by neurological disorders in some of these susceptible species including horses and humans [12], [13].

Turkey, located in the northeastern part of the Mediterranean region, is considered as a potentially endemic zone for several arthropod-borne viral infections, including WNV, due to suitable ecological and climatic conditions [14]. A considerable body of evidence that demonstrate the presence and activity of WNV in Turkey have accumulated. Serological surveillance data suggested human and animal exposure to WNV in some provinces, and an outbreak in 2010 involving individuals as well as sporadic cases since 2009, indicate symptomatic infections [15]–[17]. A recent report also revealed WNV infection in mosquitoes capable of virus transmision to mammalian species in a region neighboring Greece, the site of a concominant WNV outbreak in 2010 [18]. However, current information on virus epidemiology and expansion is only preliminary, with cross-sectional data originating from localized, dissipated regions. Moreover, definitive information on WNV activity is lacking in several regions harboring probable vector activity [14]. This study was undertaken to investigate the frequency and distribution WNV infection in vector mosquitoes and several mammal and avian species encompassing a large geographical area, to better understand virus epidemiology and to predict regions with higher risk for exposure that require established surveillance.

## Methods

### Study Design

**Figure 1:**
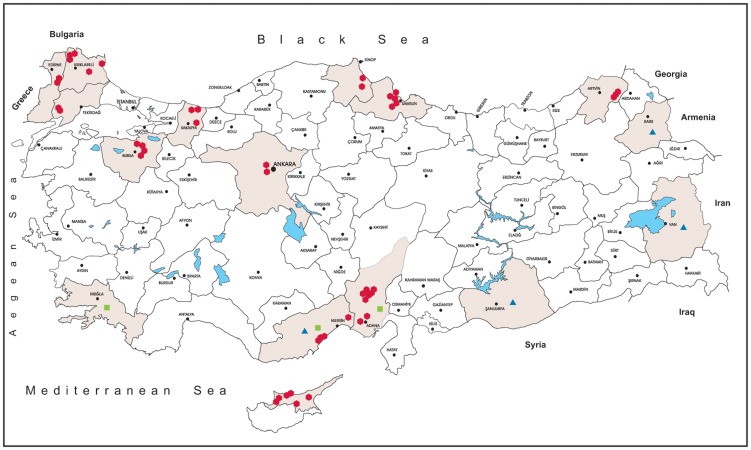
Illustrative map of provinces targeted for sampling in the study. (red circle: entomological screening, blue triangle: serological screening, green square: Viral RNA screening.)

The study was conducted in 15 provinces, distributed across Turkey, covering 21.6% of the land area (175.918/814.578 km^2^) of the country and in 4 districts of the Turkish Republic of Northern Cyprus, covering 76.6% of the land area (2.570/3.355 km^2^) (**Figure 1**).

### Ethics Statement

The study and associated protocols were designed based on national ethical legislative rules and approved by Local Ethic Committees of Mersin University (Nr: MULEC/01.09.10; for human cases) and Ankara University (Nr: AULEC/201-96-346; for animal samples). All samples were collected after written informed consent of the individuals (blood donors), according to the updated version of the Declaration of Helsinki (Seul, 2008), as indicated in the aforementioned agreements. All animal samplings were conducted based on the national regulations on the operation and procedure of animal experiments ethics committees (Regulation Nr.26220, Date:09.7.2006). Written informed consent of animal owners were also carried out prior to sampling.

### Serological Screening of WNV Exposure in Animals

WNV exposure was evaluated in sera obtained from horses, sheep and ducks from Sanliurfa, Van and Kars provinces as well as human plasma samples from Mersin province, via the detection of specific neutralizing antibodies by plaque reduction neutralization test (PRNT). In PRNT, WNV strain NY99-4132 and Vero cells (ATCC CCL81) were employed and the test was performed as previously described with minor modifications [19]. Briefly, 0.1 mL diluted serum (1∶10) was inactivated at 56°C for 30 min and mixed with an equal volume of virus in Dulbecco's modified Eagle's medium containing 5% fetal calf serum to produce an estimated 100 plaque-forming units of virus per 0.2 mL. Virus-antibody mixtures were then inoculated as 0.2 mL volumes onto Vero cell monolayers in 12-well plates and overlaid with 3.2% carboxymethyl cellulose in 2× Dulbecco's modified Eagle's medium. Plaques were scored on 4th day following incubation at 37°C. Sera that produced 90% neutralization of the challenge virus (PRNT_90_) were considered reactive. Convescelent sera from PCR-confimed WNV exposure in an equine and human subject were employed as positive controls. All experiments were performed in duplicate.

### Mosquito Sampling and Processing

Field sampling of mosquitoes was performed in 11 provinces in Turkey and at 4 provinces in the Turkish Republic of Northern Cyprus, during May-September from 2011 to 2013 (**Figure 1** **, Table S1**). Sampling was carried out in Adana and Mersin provinces in 2011; in Adana, Ankara, Edirne and Tekirdag provinces in 2012; in Artvin, Bursa, Edirne, Kirklareli, Sakarya, Samsun, Sinop, Tekirdag provinces and in Lefkosa, Girne, Magosa and Guzelyurt provinces of Cyprus in 2013. A total of 92 sites at 46 locations in suburban environments around villages were sampled using CDC Miniature Light Traps (John W. Hock Company, Gainesville, FL, USA). Furthermore, mouth aspirators were employed for collecting adult mosquitoes from inside and outside houses and barns at each site. Light traps were placed 1–2 meters above ground in peridomestic sites and left on site from 18:00 to 06:00 each night. Captured mosquitoes were collected next morning, kept alive and transferred on ice. The morphological identification of the captured mosquitoes to species level was accomplished by using published keys [20], [21]. In individual *Culex* specimens, which were either damaged to prevent identification (denoted as *Culex spp.*) or observed to belong in *Culex pipiens* sensu lato complex, legs were dissected and kept in 95% ethyl alcohol. Subsequently, all specimens were pooled according to the collection site and species to include 1–20 individuals regardless of sex, and stored at −80°C. Mosquito pools were homogenized as described previously and clarified by centrifugation at 4000 rpm for 4 minutes, prior to nucleic acid purification [22].

### Qualitative and Quantitative Detection of WNV Nucleic Acids

Plasma samples from asymptomatic blood donors and horses from Mersin province, horses from Adana and Mugla provinces and mosquito pool supernatants were used for WNV RNA detection. Each sample was subjected to nucleic acid purification by High Pure Viral Nucleic Acid Kit (Roche Diagnostics, Mannheim, Germany), followed by reverse transcription via random hexamer primers using RevertAid First Strand cDNA Synthesis Kit (Thermo Scientific, Tokyo, Japan). WNV RNA was sought via two specific polymerase chain reaction (PCR) assays; a nested PCR targeting E protein-coding region and an in-house quantitative one-step real-time Reverse Transcription (rRT) PCR, targeting 3′ non-coding region in the viral genome, as described previously [18], [23]. Amplicons obtained from Vero cell culture grown NY99-4132 strain were cloned in pTZ57R plasmid via T4 DNA Ligase (Thermo Scientific, Tokyo, Japan), quantitated spectrophotometrically and used as standards in 10-fold dilutions in the rRT-PCR assay, carried out using QuantiTect Probe RT-PCR kit (Qiagen, Germany) in a Rotor-Gene 6000 instrument (Corbett Research, Australia). The nested PCR amplicons of the expected size of 248 basepairs were detected under ultraviolet light after electrophoresis in 1.7% agarose gels. In positive samples, amplicons of the nested PCR were purified using High Pure PCR Product Purification Kit (Roche Diagnostics, Mannheim, Germany) and subjected to nucleotide sequencing with forward and reverse amplification primers.

### DNA Barcoding in Mosquitoes

A 658-bp sequence of the cytochrome c oxidase I (COI) gene, widely used for biological barcoding, was amplified in WNV-positive mosquito pools, composed of specimens identified morphologically as *Culex* spp. or as *Culex pipiens* sensu lato, for further characterization [24]. Dissected legs from individual mosquitoes, stored in 95% ethyl alcohol, were processed with DNeasy Blood & Tissue Kit (Qiagen, Hilden, Germany) and amplified with primers LCO1490 and HCO2198 as described previously [24]. PCR products were cleaned up using High Pure PCR Product Purification Kit (Roche Diagnostics, Mannheim, Germany) and subjected to nucleotide sequencing with forward and reverse primers.

### Sequencing and Phylogenetic Analysis

Nested PCR amplicons from all WNV positive mosquito pools, viremic animals and COI amplicons from mosquito specimens were characterized by sequencing using an ABI Prism 310 Genetic Analyzer (Applied Biosystems, Foster City, CA, USA). The sequences obtained were aligned and analyzed using CLC Main Workbench v5.5 (CLCBio, Aarhus, Denmark) and subsequently, by MEGA software v5.2 [25]. Phylogenetic trees were constructed using the Jukes-Cantor substitution rate model with 500 bootstrap replicates. Maximum likelihood trees were generated based on the Unweighted Pair Group Method with Arithmetic Mean (UPGMA) tree.

## Results

### WNV Neutralizing Antibodies in Humans and Animals

A total of 1180 sera, obtained from horses (389, 42.6%) from Sanliurfa and Van provinces, ducks (423, 46.2%) from Kars province, sheep (102, 11.2%) from Sanliurfa province and humans (266, 22.5%) from Mersin province were included in the PRNT. Neutralizing antibodies were detected in 124 samples, and overall WNV seroprevalance was calculated as 10.5%. Due to the suboptimal storage and transport conditions, the samples were not included in viral RNA investigations, except for human samples from Mersin province. Seropositivity rates according to the species and sampling locations are provided in **Table 1**.

**Table 1:** WNV neutralizing antibody and nucleic acid detetion rates according to sampling location and species.

Location	Species	Number of samples	PRNT Positive
Kars province	Duck	423	42 (9.9%)
Sanliurfa province	Horse	218	30 (13.8%)
	Sheep	102	2 (1.9%)
Van province	Horse	171	18 (10.5%)
Mersin province	Human	266	32 (12.1%)
***Total***		**1180**	**124 (10.5%)**
			**RNA Positive**
Adana province	Horse	121	6 (4.9%)
Mersin province	Horse	73	6 (8.2%)
	Human	266	0 (0%)
Mugla province	Horse	62	19 (30.6%)
***Total***		**522**	**31 (5.9%)**

### WNV Nucleic Acids in Human and Equine Samples

**Figure 2:**
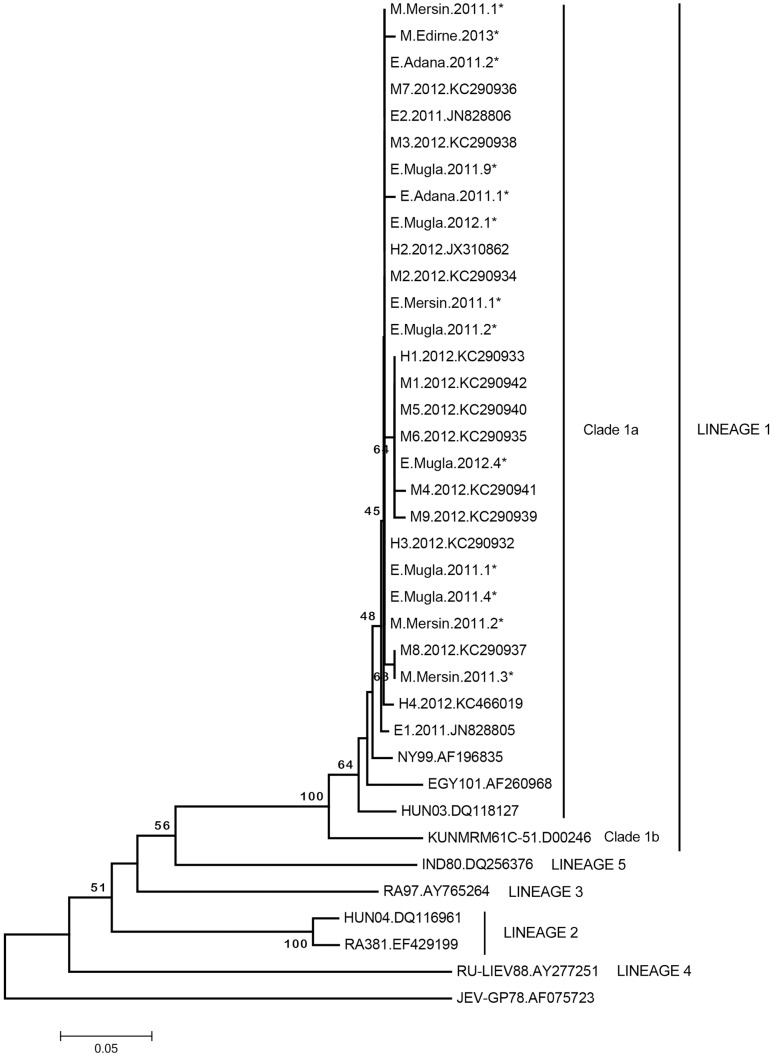
Neighbor-joining analysis of partial West Nile virus sequences. Viruses included in the analysis are indicated with isolate name and GenBank accession number. Japanese Encephalitis Virus (JEV) isolate GP78 is employed as an outlier. Sequences characterized in the study are marked with an asterisk and indicated with host, location, year and number (E: equine, M: mosquito, H: human sequences).

WNV RNA was investigated in 522 samples that comprise 266 human plasma (50.9%) from Mersin province and 256 equine plasma (49.1%) from Mersin, Adana and Mugla provinces. Viremia was detected in a total of 31 samples (31/522, 5.9%), via nested and rRT-PCR assays. All samples with positive WNV RNA originated from equines without clinical symptoms and no viremic human sample was noted. Due to the limited amounts of available material, WNV PRNT was not performed in these samples. Viral RNA detection rates according to the species and sampling locations are provided in **Table 1**. Viral loads determined in positive samples were relatively low, observed within 10^2^ to10^4^ copies/mL range. Amplicons of nested PCR were sequenced and characterized in all positive samples (GenBank accession numbers: KJ433827-KJ433836) All sequences were grouped with WNV lineage 1 clade 1a isolates, and demonstrated 0–17% intramural divergence in the maximum composite likelihood analyses (**Figure 2** **, Table S2**). Partial sequences with the highest variation rates were detected in the same location, and in different years (2011 and 2012). Comparison of the current sequences with those obtained from horses with neurological disease from Central Anatolia in 2011 revealed 3–17% nucleotide divergence (**Table S2**). Three sequences characterized in Mugla province were identical to patient-derived sequences from Ankara (Central Anatolia) and Tekirdag (Eastern Thrace) provinces, as well as mosquito-derived sequences from Eastern Thrace in 2012. Otherwise, overall similarities of 72.29–99.60% and 84–97.6% were observed among sequences identified previously in Turkey and from lineage 1 strains from Hungary, Egypt and New York, respectively (**Table S2**).

#### Species distribution and WNV detection in mosquitoes

A total of 2642 mosquito specimens belonging to 15 species were captured in locations in Turkey and Northern Cyprus, which comprise *Ochlerotatus caspius* (1385, 52.4%), *Cx. pipiens* s. l. (640, 24.2%), *Cx. theileri* (203, 7.7%), *Anopheles maculipennis* s. l. (166, 6.3%), *Coquillettidia richiardii* (114, 4.3%), *Culex* spp. (47, 1.8%), *Dahliana geniculata* (36, 1.4%), *Cx. tritaeniorhynchus* (14, 0.52%), *Cx. torrentium* (14, 0.52%), *Cx. pusillus* (7, 0.3%), *An. superpictus* (6, 0.2%), *An. claviger* (3, 0.1%), *Culiseta longiareolata* (3, 0.1%), *Cs. annulata* (2, 0.1%), *Aedes vexans* (1, 0.04%) and *Oc. pulcritarsis* (1, 0.04%). The number and distribution of mosquito species according to the sampling locations are provided in **Table 2**.

**Table 2:** Distribution of mosquito species according to the sampling location.

Species	Number of Samples												
	Adana	Ankara	Artvin	Bursa	Edirne	Mersin	Kirklareli	Sakarya	Samsun	Sinop	Tekirdag	N.Cyprus	Total
*Oc. caspius*	-	-	2	-	1164	-	3	-	19	3	194	-	1385 (52.4%)
*Cx. pipiens s.l.*	256	18	28	10	3	108	3	85	44	-	38	48	640 (24.2%)
*Cx. theileri*	-	-	9	172	22	-	-	-	-	-	-	-	203 (7.7%)
*An. maculipennis s.l.*	-	2	-	-	9	-	1	147	-	-	7	-	166 (6.3%)
*Cq. richiardii*	100	-	-	-	-	7	3	4	-	-	-	-	114 (4.3%)
*Culex spp.*	8	-	-	19	20	-	-	-	-	-	-	-	47 (1.8%)
*Da. geniculata*	-	-	-	2	1	-	33	-	-	-	-	-	36 (1.4%)
*Cx. tritaeniorhynchus*	4	-	-	-	-	10	-	-	-	-	-	-	14 (0.52%)
*Cx. torrentium*	13	-	-	1	-	-	-	-	-	-	-	-	14 (0.52%)
*Cx. pusillus*	-	-	-	-	-	7	-	-	-	-	-	-	7 (0.3%)
*An. superpictus*	6	-	-	-	-	-	-	-	-	-	-	-	6 (0.2%)
*An. claviger*	-	-	-	-	2	-	-	1	-	-	-	-	3 (0.1%)
*Cs. longiareolata*	2	-	-	1	-	-	-	-	-	-	-	-	3 (0.1%)
*Cs. annulata*	-	-	-	-	-	-	-	1	-	-	-	-	2 (0.1%)
*Ae. vexans*	-	-	1	-	-	-	-	-	-	-	-	-	1 (0.04%)
*Oc. pulcritarsis*	-	-	-	1	-	-	-	-	-	-	-	-	1 (0.04%)
Total	389 (14.7%)	20 (0.8%)	40 (1.5%)	206 (7.8%)	1221 (46.2%)	132 (5.0%)	43 (1.6%)	238 (9.0%)	63 (2.4%)	3 (0.1%)	239 (9.1%)	48 (1.8%)	2642 (100%)

Captured mosquitoes were distributed into 203 pools according to the collection site and species and tested for WNV RNA. A total of 4 pools (1.9%) were positive in nested and rRT-PCR assays, with viral loads ranging from 10^4^ to 10^7^ copies/mL. WNV-positive pools originated from Edirne and Mersin provinces and included *Oc. caspius* and *Cx. pipiens s.l.* mosquitoes, respectively (**Table 3**). Partial characterization of the amplicons revealed all isolates to belong in WNV lineage 1 clade 1a strains (GenBank accession numbers: KJ433837-KJ433840). Limited diversity (1–2%) was noted among positive pools and several previous human and mosquito-derived sequences from Central Anatolia and Eastern Thrace were identical to those detected in pools, as well as in horses from Mugla province, characterized in this study. Nevertheless, 1–18% divergence was also noted between positive pools and other sequences from Turkey and similarities of 94.8–96.8% were observed, with WNV lineage 1 strains of the Old and New Worlds (**Figure 2**, **Table S2**).

**Table 3:** Features of mosquito pools positive for WNV RNA.

Pool No.	Sampling Location	Sampling Date	Sequence ID	Morphological Identification	DNA Barcoding
1	Mersin province; Location E1 (36°42′21.4760″, 034°22′22.7520″)	1–3 September, 2011	WNV lineage 1 (KJ433838)	*Cx. pipiens s.l.* (1 specimen)	Not conclusive
2	Mersin province; Location E2 (36°42′17.0840″, 034°22′22.8300″)	1–3 September, 2011	WNV lineage 1 (KJ433839)	*Cx. pipiens s.l.* (1 specimen)	Not conclusive
3	Mersin province; Location E2 (36°42′17.0840″, 034°22′22.8300″)	1–3 September, 2011	WNV lineage 1 (KJ433840)	*Cx. pipiens s.l.* (7 specimens)	*Cx. quinquefasciatus* (6/7) *Cx. perexiguus* (1/7)
4	Edirne province; Location B3 (41°15′57.6060″, 026°47′55.1222″)	5–7 July, 2013	WNV lineage 1 (KJ433837)	*Oc. caspius* (14 specimens)	Not performed

**Figure 3:**
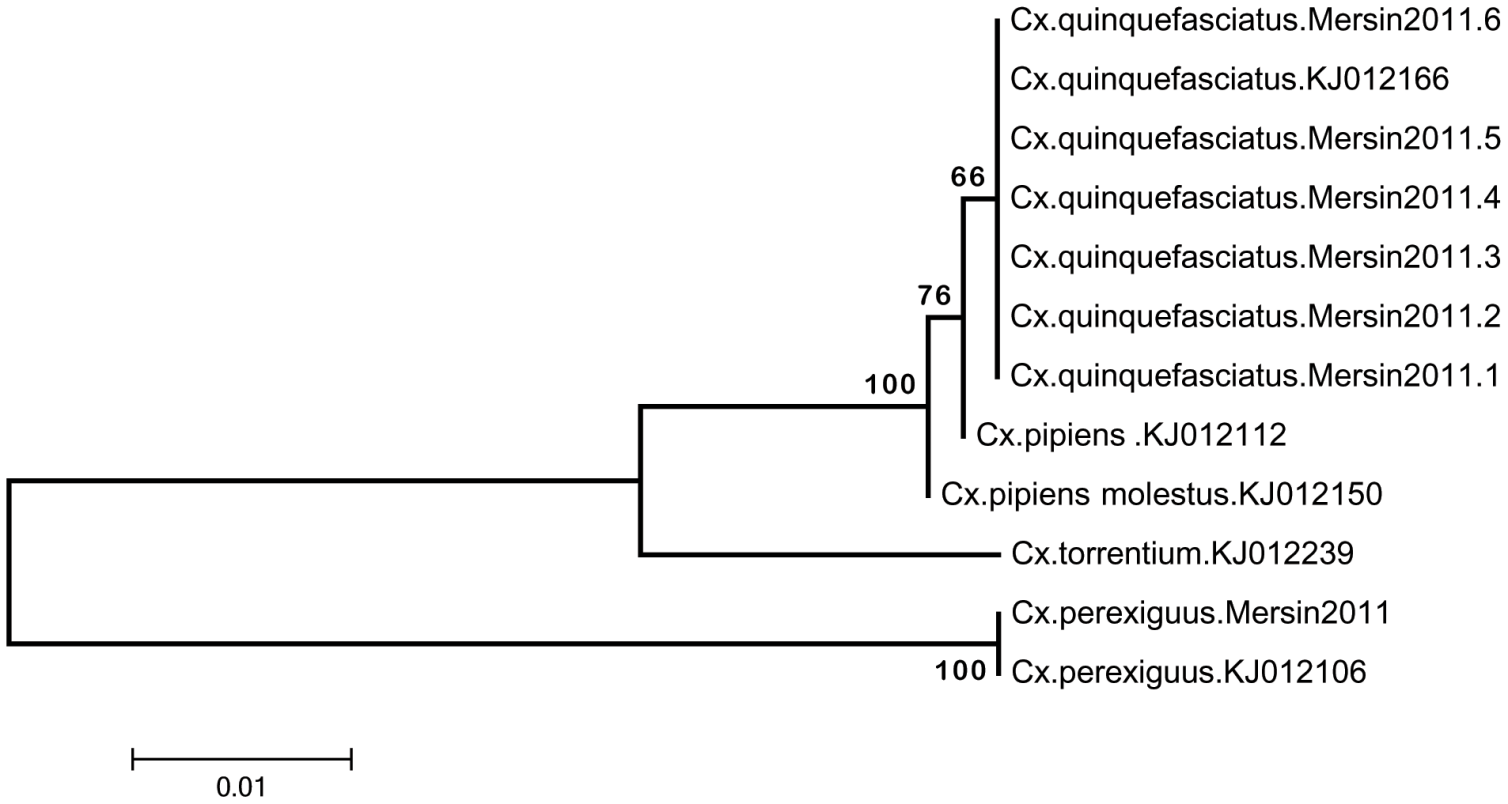
Neighbor-joining analysis of partial cytochrome c oxidase I gene sequences obtained from ethanol-stored legs from individual mosquitoes in WNV positive pools. Sequences characterized in the study are given as organism, location and year whereas standard sequences are indicated as organism and GenBank accession number.

COI PCR and sequence analysis were performed in ethanol-stored dissected legs from individual mosquitoes of WNV positive pools, identified morphologically as *Cx. pipiens* sensu lato. In two pools, that comprise single individuals, COI PCR did not provide optimal amplicons for sequencing. However, in one pool, individual mosquitoes revealed sequences of *Cx. quinquefasciatus* and *Cx. perexiguus* species, from *Cx. pipiens* complex (**Table 3**) (GenBank accession numbers: KJ012171, KJ012162, KJ012163, KJ012170, KJ012168, KJ012169 and KJ012103, respectively). Phylogenetic analysis further confirmed the identity of these individuals (**Figure 3**).

## Discussion

Current evidence indicates that WNV is expanding its geographical range in Europe as well as in the rest of the world, causing increasing numbers of outbreaks associated with human morbidity and mortality. Multiple introductions of various WNV strains has been demonstrated in Europe and surrounding regions, raising concerns about the potential emergence of strains with increased virulence [1]. Turkey, a contiguous transcontinental country, located mostly on Anatolia in Western Asia, and on Eastern Thrace in Southeastern Europe, provides suitable habitats for WNV introduction and persistence [14]. The present study was carried out to confirm and complement the currently existing information on WNV circulation in Turkey. It covers a large geographical area including regions with preliminary data as well as previously unexplored locations including the Turkish Republic of Northern Cyprus. Furthermore, it involves screening of mosquitoes and asymptomatic infections in avian and several mammal species including humans and horses with significant number of samples. To our knowledge, this is the most comprehensive study undertaken in Turkey to identify WNV activity and characterize circulating viruses [14].

For the detection of previous WNV exposure, the presence of neutralizing antibodies was sought via PRNT by in 1180 sera originating from humans, horses, sheep and ducks in the study. Serological screening was performed in samples from Mersin, Sanliurfa, Van and Kars provinces, located in southern-eastern Anatolia, with very limited available virus-specific data on WNV circulation [14]. Here, an overall rate of 10.5% with a 12.5% human seroprevalence was observed (**Table 1**). The only available animal serosurveillance data from the region originates from Sanliurfa province in 2006, prior to the emergence of WNV in Turkey during 2010, where WNV neutralizing antibodies were detected in 4% of cattle sera tested [26]. Moreover, human WNV exposure in 9.4% and 17% were reported from Siverek district of Sanliurfa province and Kiziltepe district of Mardin province, respectively [27], [28]. The current data demonstrate the circulation of WNV not only in south-southeast Anatolia but indicates a previously unexplored region of WNV activity in Eastern Anatolia, neighboring Iran, Armenia and Georgia. A previous animal serosurvey undertaken in Hatay, Adana, Antalya (Mediterranean), Mugla, Izmir (Aegean), Bursa (Northwest Anatolia) and Ankara provinces (Central Anatolia) had also revealed the presence of WNV neutralizing antibodies in a variety of mammalian species, including 2.5% of ass-mules, 4% of cattle, 37.7% of dogs, 13.5% of horses, 1% of sheep and 20.4% of humans [26]. Moreover, human WNV seroreactivity rates of 0.56–21.5% were reported from Central Anatolia, Aegean and Eastern Thrace regions [14], [17]. While a direct comparison among species and regions may be misleading due to the dynamic interplay of various factors including local climate and mosquito distribution, these results indicate the occurence of WNV infections in a wide range of mammals and domestic avians which can contribute to the long-term virus survival in the absence of overt disease.

To provide further evidence for virus circulation in susceptible mammals, WNV nucleic acids were investigated in samples collected during the mosquito season in Mugla, Mersin and Adana provinces of Aegean-Mediterranean Anatolia during 2011–2012. Nested and quantitative rRT-PCR assays, that enable sensitive detection of WNV lineages 1 and 2 were employed in screening. Viral RNA was detected in horses in all locations with an overall rate of 5.9% and a significantly higher rate of positivity was observed in Mugla province (30.6%) (**Table 1**). No positive human sample was noted. WNV is known to be transmitted to equids by bridge vectors and is responsible for the majority of equine flaviviral encephalitis worldwide [29]. Similar to that in humans, most horses seroconvert without clinical disease after exposure to WNV. In approximately 8% of naive horses, severe WNV disease with neurological symptoms develop and the incidence of disease in equids has increased significantly since the mid-1990s, in parallel with the dispersion of lineage 1a WNV strains [30], [31]. Since 2010, WNV infections among horses were repeatedly reported in the Mediterranean basin and other European countries, such as in Bulgaria, Croatia, Greece, Italy, Macedonia, Morocco, Portugal, Romania and Spain [29], [32]. Equine encephalitis due to WNV lineage 1a in Turkey with a favorable outcome has been initially described in Eskisehir province (Central Anatolia) in 2011 [16]. Despite detection of equine exposure via neutralizing antibodies in Bursa, Adana and Eskisehir provinces, WNV RNA has not been characterized in asymptomatic horses or in other mammal/avian species previously in Turkey [16], [26], [33]. Horses are considered as dead-end hosts for WNV due to the short and low magnitude viremia observed following infected mosquito bites. A maximum serum virus titer of 10^3^ pfu/ml and viremia duration of 6 days have been demonstrated to occur in experimental equine infections with WNV, which is unlikely to initiate an infection in the midgut epithelia of the mosquito vector [29], [34], [35]. Since RNA levels detected via rRT-PCR in positive equine plasma this study are low and roughly correspond to the levels noted in experimental infections, it can be assumed that they do not significantly contribute to the virus lifecycle by infecting vectors. However, the RNA detection in horses in subsequent years (2011 and 2012) reveals ongoing WNV activity in the region, which is further supported by the findings of the field study for vectors.

Entomological surveillance performed at 11 provinces of Turkey and at 4 provinces of the Turkish Republic of Northern Cyprus revealed *Cx. pipiens* s. l. to be the most abundant mosquito species in Adana, Ankara, Artvin, Mersin and Samsun provinces as well as in Cyprus (**Table 2**). However, specimens belonging to 15 different species were captured, with the predominance of *Oc. caspius* in Edirne and Sinop provinces, *Cx. theileri* in Bursa province and *Da. geniculata* in Kirklareli province. It is known that different mosquito species demonstrate variable competencies to support WNV replication and transmission. Mosquitoes belonging in *Culex* species, from which initial WNV isolates were recovered, are widely accepted as the primary global transmission vector [13]. Several *Culex* species have been shown to be competent for WNV transmission in North America and *Cx. pipiens* s.s., *Cx. perexiguus*, and *Cx. modestus* are considered as important vector species in Europe [36]–[38]. Moreover, WNV has also been detected in other genera of mosquitoes including *Ochlerotatus*, *Aedes*, *Anopheles*, *Coquillettidia*, *Aedeomya*, *Mansonia*, *Mimomyia*, *Psorophora*, *Culiseta* and *Uranoteania*, which serve as bridge vectors critical to transmission from birds to humans and equines [38]. *Cx. pipiens* is known to be widespread in the Anatolian fauna, with many other mosquito species that can serve as bridge vectors [39]. Species distribution observed in this study further confirmes this data and maintains the presence and abundance of several mosquito species capable of transmitting WNV to various avian and mammals including equines and humans. However, since the sampling could not be carried on during the whole mosquito season in any given sampling location, the species frequencies and distribution reported in this study represent cross-sectional data for each region.

Field-captured mosquitoes were pooled and screened for WNV infection using the same approach for detecting nucleic acids in animal plasma. Viral RNA was identified in 1.9% of 203 pools and characterized as WNV lineage 1 (**Table 3**). Three of the positive pools originated from two sampling sites of the same location in Mersin province, and has been collected during early September, 2011. To confirm the identity of the mosquitoes in the infected pools, DNA barcoding via COI sequence analysis was performed in dissected legs from individual mosquitoes from each pool. Despite the lack of succesful amplification in two individual mosquitoes comprising two infected pools, *Cx. quinquefasciatus* and *Cx. perexiguus* mosquitoes were identified in another WNV positive pool (**Table 3** **,** **Figure 3**). Detection and verification of these specimens as *Cx. quinquefasciatus* comprises the first record of the species in Turkey. *Cx. quinquefasciatus*, also called the southern house mosquito, is an important WNV vector in the southern United States as well as in Africa [12], [40]. *Cx. perexiguus* is another highly-competent WNV vector, participating not only in enzootic cycle of the virus but also in transmission to equines [38], [41]. This individual mosquito, confirmed as *Cx. perexiguus* in DNA barcoding, is likely to be misidentified as *Cx. pipiens s.l.* during morphological evaluation. Identification of these species in WNV-infected pools in Turkey is a novel finding, revealing the activity of many competent WNV vectors in the country. Another infected pool comprised *Oc. caspius* specimens collected from Edirne province, Eastern Thrace region in 2013. Interestingly, the initial detection of WNV sequences in vectors was accomplished in this region during 2012, in *Cx. pipiens* s.s. and *Oc. caspius* pools [18]. Thus, our current findings verify and demonstrate an ongoing circulation of WNV in mosquitoes in this region. In Turkey, other efforts to detect WNV in potential mosquito vectors in regions with evidence for virus activity has been unsuccessful. For example, WNV antigens or nucleic acids could not be detected in mosquito specimens belonging in *Cx. pipiens* s.l., *Oc. caspius*, and *Aedes* species captured in Sanliurfa province, despite serological evidence of virus exposure in the region [42]. Likewise, a recent field survey undertaken in Ankara province Central Anatolia, where symptomatic WNV cases have been demonstrated, *Cx. pipiens* s.l., *An. maculipennis* and *An. claviger* species were collected but WNV infection in vectors could not be identified [43]. Extensive surveillance activities are required for these regions, to reveal and characterize WNV infection in vectors. In Europe, several reports indicate *Cx. pipiens* s.l., *Cx. modestus*, *Oc. caspius* and *Cx. perexiguus* as the most common species associated with WNV infections and the potential involvement of *Cx. univittatus, Cx. theileri* and *An. maculipennis* s.l. in virus circulation [3], [38]. Furthermore, in the Volgograd region of Russia and in Israel, *Cx. pipiens*, *Cx. modestus*, *Cx. perexiguus* and *Oc. caspius* species were implicated in WNV transmission [44], [45]. Accurate identification of mosquito species in a region is of significant importance to reveal and predict WNV emergence, since only certain species in any given area can act effectively as primary and bridge vectors to human or equine populations. Evidence from Italy, Spain and Greece suggests that WNV detection in mosquitoes precedes the appearance of human or equine cases and surveillance activities provide crucial information on the relevant vectors and on the circulating virus strain, for optimal diagnostic procedures and interventions for preventing transmission [38]. Another example is provided from Turkey, where infected mosquitoes could be detected approximately 4 weeks prior to the emergence of human cases in the Eastern Thrace region [17], [18]. Thus, monitorization of WNV activity via vector and/or sentinel animal surveillance is required for Turkey, especially for regions with evidence for virus exposure and insufficient data on vectors.

WNV strains are grouped in several putative genetic lineages [9], [11]. Isolates belonging to lineage 1 are widely distributed and highly invasive, and account for the majority of the strains responsible for the European and the Mediterranean Basin outbreaks [3], [46]. However, lineage 2 strains, which have recently spread from Austria and Hungary to Balkan States and Greece, have also emerged and caused outbreaks resulting in human and bird mortality, particularly in Greece [47]. In Italy, co-circulation of lineage 1 and 2 strains has also been demonstrated [38]. At least five new lineages (lineages 3–7) have been proposed for strains isolated in central Europe, as well as in Russia and India [11]. All WNV genomic data obtained in Turkey, including partial E gene sequences characterized from viremic horses and mosquito pools in this study, are grouped with WNV lineage 1 clade 1 strains (**Figure 2**). Overall, maximum composite likelihood analyses revealed similarities among regions and hosts, as well as identical sequences observed in Mugla province in horses during 2011 to *Cx. pipiens s.l.* pools in Mersin province collected during the same year (**Table S2**). Moreover, current sequences displayed limited diversity compared to previously-obtained data and sequences identical with patient and vector-derived sequences from Ankara, Tekirdag and Edirne provinces in 2012 were also noted, despite geographical and temporal separation (**Table S2**). These data suggest that WNV strains in circulation are generally genetically conserved with restricted sequence diversity among strains. Nevertheless, the origin and variability in WNV strains in Turkey will be better elucidated upon whole genome sequencing of the isolates, for which studies are underway by our group.

In conclusion, our findings from vectors and exposed animals indicate a wider zone of WNV activity in Turkey than previously anticipated, including Eastern Thrace and Mediterranean-Aegean regions as well as Southeastern and Northeastern Anatolia. Ongoing virus circulation with limited genomic diversity was observed in certain regions. WNV must be considered in etiology of human or equine febrile diseases with/without central nervous system involvement in these regions. Zones of priority for surveillance of WNV activity via mosquito and/or sentinel animals must be established.

## Supporting Information

Table S1 List of mosquito sampling location and sites employed in the study.
(DOCX)

Table S2 Pairwise nucleotide diversity (above diagonal) and genetic similarity (below diagonal) among E protein-coding genome segment of WNVs identified in Turkey.
(DOCX)
