# Supplementary material for: Serological, Molecular and Entomological Surveillance Demonstrates Widespread Circulation of West Nile Virus in Turkey
Source: PLoS Negl Trop Dis. 2014 Jul 24;8(7):e3028. doi: 10.1371/journal.pntd.0003028 (PMC4109882; doi:10.1371/journal.pntd.0003028)
Supplement: Table S1 — List of mosquito sampling location and sites employed in the study. (DOCX) [file pntd.0003028.s001.docx]

**Supplementary Table 1:** List of mosquito sampling location and sites employed in the study

| **Location** | **Site** | **Coordinates** | **Altitude** |
| --- | --- | --- | --- |
| Adana province | C1 | 37°20'21.7842", 035°36'39.1022" | 175 |
|  | C2 | 37°20'30.1912", 035°36'34.4770" | 178 |
|  | C3 | 37°20'31.4747", 035°36'29.2393" | 180 |
|  | C4 | 37°20'36.9703", 035°36'38.9375" | 188 |
|  | C5 | 37°20'47.3434", 035°36'49.5774" | 178 |
|  | D1 | 37°22'56.6634", 035°37'56.3484" | 205 |
|  | D2 | 37°23'03.3160", 035°38'02.0695" | 201 |
|  | D3 | 37°22'59.9087", 035°38'09.3899" | 185 |
|  | D4 | 37°22'58.6164", 035°38'10.6049" | 185 |
|  | D5 | 37°22'56.9741", 035°38'10.0595" | 187 |
|  | I1 | 37°15'34.5756", 035°39'45.6962" | 80 |
|  | K1 | 37°17'21.3102", 035°39'10.8860" | 103 |
|  | K2 | 37°17'19.7306", 035°39'11.8862" | 105 |
|  | K3 | 37°17'19.3694", 035°39'00.5047" | 108 |
|  | O1 | 37°18'07.2787", 035°31'05.6633" | 236 |
|  | O2 | 37°18'07.6206", 035°31'06.3651" | 236 |
|  | O3 | 37°18'10.0243", 035°31'04.6991" | 228 |
|  | T1 | 37°21'45.7947", 035°37'04.5708" | 173 |
|  | T2 | 37°21'56.0508", 035°37'10.2112" | 161 |
|  | T3 | 37°21'57.6446", 035°37'10.0228" | 162 |
|  | T4 | 37°21'57.8526", 035°37'09.4203" | 163 |
|  | T5 | 37°21'51.3791", 035°37'29.2325" | 159 |
|  | Z1 | 37°24'08.1418", 035°37'54.3769" | 238 |
|  | Z2 | 37°24'21.0353", 035°37'52.2662" | 257 |
|  | Z3 | 37°24'21.3846", 035°37'52.6037" | 255 |
|  | Z4 | 37°24'18.6589", 035°37'54.0943" | 251 |
|  | H | 36°59'23.1400", 035°13'50.9500" | 3 |
|  | A | 37°01'33.9100", 035°20'25.7300" | 17 |
| Ankara province | I1 | 39°54'09.7900", 032°54'19.5800" | 910 |
|  | I2 | 39°54'11.8400", 032°54'07.2300" | 892 |
|  | Y | 39°50'01.0200", 032°56'46.1600" | 1381 |
| Artvin province | Y | 40°46'56.0284", 041°30'16.1563" | 425 |
|  | A | 41°07'15.8676", 042°06'25.1175" | 970 |
|  | T | 41°23'34.1164", 041°41'37.3391" | 110 |
| Bursa province | Y1 | 40°11'24.9659", 029°24'14.5611" | 486 |
|  | Y2 | 40°11'25.8119", 029°24'14.4218" | 453 |
|  | Y3 | 40°11'27.7053", 029°24'16.9413" | 472 |
|  | G1 | 40°20'02.5982", 029°01'39.1952" | 353 |
|  | G2 | 40°20'07.2662", 029°01'50.3957" | 397 |
|  | G3 | 40°20'07.9493", 029°01'39.1859" | 353 |
|  | C1 | 40°10'36.8939", 029°10'30.7241" | 337 |
|  | H | 40°10'31.6572", 029°11'21.4168" | 334 |
| Edirne province | B1 | 41°36'54.8072", 026°57'58.2783" | 100 |
|  | B2 | 41°15'57.8929", 026°47'52.2847" | 101 |
|  | B3 | 41°15'57.6060", 026°47'55.1222" | 95 |
| Mersin province | M | 36°45'48.4200", 034°30'20.6400" | 89 |
|  | E1 | 36°42'21.4760", 034°22'22.7520" | 102 |
|  | E2 | 36°42'17.0840", 034°22'22.8300" | 166 |
|  | E3 | 36°42'17.3240", 034°22'22.8300" | 166 |
|  | G | 36°58'04.0783", 035°06'38.4090" | 34 |
| Kirklareli province | V | 41°34'20.7391", 027°45'50.8021" | 164 |
|  | K | 41°37'25.9013", 028°04'42.3620" | 25 |
|  | I1 | 41°52'31.9440", 027°58'55.6933" | 31 |
|  | I2 | 41°52'16.9327", 027°58'32.7318" | 7 |
|  | I3 | 41°51'32.0334", 027°34'04.9183" | 294 |
|  | D | 41°43'06.7813", 027°35'42.5109" | 283 |
| Sakarya province | K1 | 41°04'36.9972", 030°33'13.6532" | 121 |
|  | K2 | 41°04'48.4404", 030°33'16.5712" | 97 |
|  | K3 | 41°05'01.1868", 030°33'15.1985" | 63 |
|  | KM | 41°06'36.3623", 030°32'02.5995" | 62 |
|  | H | 41°02'10.4693", 030°51'55.7640" | 111 |
| Samsun province | D | 41°22'08.0963", 036°13'44.2140" | 8 |
|  | E1 | 41°30'32.2744", 035°59'35.5926" | 37 |
|  | E2 | 41°31'23.6458", 035°58'51.3261" | 64 |
|  | K1 | 41°29'51.1747", 035°57'58.9114" | 31 |
|  | K2 | 41°28'47.7019", 035°58'14.8972" | 133 |
|  | Y | 41°34'18.8857", 035°52'37.3392" | 27 |
|  | C | 41°03'51.1227", 036°49'23.4159" | 330 |
|  | KV1 | 41°05'25.8851", 036°02'26.4802" | 651 |
|  | KV2 | 41°05'34.2910", 036°04'04.3757" | 732 |
| Sinop province | A | 41°33'59.0759", 035°17'36.2382" | 456 |
|  | D | 41°40'35.0570", 035°19'14.7914" | 108 |
| Tekirdag province | I1 | 40°51'39.1840", 026°48'25.6989" | 158 |
|  | I2 | 40°51'39.6587", 026°48'18.6922" | 211 |
| Lefkosa province* | H | 35°13'46.5393", 033°25'07.4721" | 139 |
|  | D1 | 35°14'40.2355", 033°29'17.3098" | 143 |
|  | D2 | 35°15'03.7652", 033°29'50.3352" | 178 |
| Girne province* | C1 | 35°17'55.3330", 033°02'49.9450" | 241 |
|  | G1 | 35°20'21.0144", 033°04'06.1311" | 42 |
|  | G2 | 35°20'21.8936", 033°04'01.1799" | 51 |
|  | G3 | 35°20'25.3024", 033°04'00.5488" | 55 |
|  | K | 35°21'16.0184", 033°07'45.6885" | 23 |
|  | L1 | 35°21'05.3645", 033°08'20.9347" | 39 |
|  | L2 | 35°21'06.9917", 033°09'36.2187" | 30 |
|  | L3 | 35°21'04.2826", 033°09'47.3865" | 31 |
| Magosa province* | G1 | 35°16'07.6325", 033°43'18.7998" | 85 |
|  | G2 | 35°16'07.8482", 033°43'15.2791" | 85 |
|  | G3 | 35°16'05.7037", 033°43'21.0783" | 97 |
|  | G4 | 35°16'11.1765", 033°43'12.3796" | 90 |
|  | G5 | 35°15'48.9168", 033°43'38.1456" | 79 |
| Guzelyurt province* | B | 35°09'47.2358", 033°01'13.0118" | 118 |

**: Locations in Northern Cyprus*
