# Supplementary material for: Serological, Molecular and Entomological Surveillance Demonstrates Widespread Circulation of West Nile Virus in Turkey
Source: PLoS Negl Trop Dis. 2014 Jul 24;8(7):e3028. doi: 10.1371/journal.pntd.0003028 (PMC4109882; doi:10.1371/journal.pntd.0003028)
Supplement: Table S2 — Pairwise nucleotide diversity (above diagonal) and genetic similarity (below diagonal) among E protein-coding genome segment of WNVs identified in Turkey. (DOCX) [file pntd.0003028.s002.docx]

**Supplementary Table 2:** Pairwise nucleotide diversity (above diagonal) and genetic similarity (below diagonal) among E protein-coding genome segment of WNVs identified in Turkey

|  | **1** | **2** | **3** | **4** | **5** | **6** | **7** | **8** | **9** | **10** | **11** | **12** | **13** | **14** | **15** | **16** | **17** | **18** | **19** | **20** | **21** | **22** | **23** | **24** | **25** | **26** | **27** | **28** | **29** | **30** | **31** |
| --- | --- | --- | --- | --- | --- | --- | --- | --- | --- | --- | --- | --- | --- | --- | --- | --- | --- | --- | --- | --- | --- | --- | --- | --- | --- | --- | --- | --- | --- | --- | --- |
| **HUN03.DQ1181271** |  | 0.02 | 0.05 | 0.08 | 0.08 | 0.04 | 0.04 | 0.05 | 0.21 | 0.04 | 0.04 | 0.05 | 0.05 | 0.05 | 0.05 | 0.05 | 0.05 | 0.06 | 0.04 | 0.05 | 0.05 | 0.05 | 0.06 | 0.07 | 0.08 | 0.07 | 0.17 | 0.04 | 0.05 | 0.05 | 0.04 |
| **NY99.AF196835** | 98.00 |  | 0.04 | 0.07 | 0.07 | 0.03 | 0.02 | 0.04 | 0.20 | 0.03 | 0.02 | 0.04 | 0.03 | 0.03 | 0.04 | 0.03 | 0.04 | 0.05 | 0.02 | 0.03 | 0.04 | 0.03 | 0.05 | 0.08 | 0.06 | 0.05 | 0.16 | 0.02 | 0.04 | 0.03 | 0.03 |
| **EGY101.AF260968** | 94.80 | 96.00 |  | 0.08 | 0.09 | 0.05 | 0.04 | 0.05 | 0.21 | 0.05 | 0.04 | 0.05 | 0.05 | 0.05 | 0.06 | 0.05 | 0.06 | 0.06 | 0.04 | 0.05 | 0.06 | 0.05 | 0.07 | 0.09 | 0.08 | 0.07 | 0.18 | 0.04 | 0.05 | 0.05 | 0.05 |
| **E1.2011.JN828805** | 92.00 | 93.20 | 92.40 |  | 0.01 | 0.06 | 0.05 | 0.03 | 0.17 | 0.06 | 0.05 | 0.04 | 0.06 | 0.06 | 0.06 | 0.05 | 0.04 | 0.05 | 0.05 | 0.05 | 0.06 | 0.04 | 0.05 | 0.03 | 0.03 | 0.07 | 0.17 | 0.05 | 0.04 | 0.05 | 0.06 |
| **E2.2011.JN828806** | 92.00 | 93.20 | 91.60 | 99.17 |  | 0.06 | 0.05 | 0.03 | 0.16 | 0.06 | 0.05 | 0.04 | 0.06 | 0.06 | 0.06 | 0.05 | 0.04 | 0.05 | 0.05 | 0.05 | 0.06 | 0.04 | 0.05 | 0.03 | 0.03 | 0.07 | 0.17 | 0.05 | 0.04 | 0.05 | 0.06 |
| **H1.2012.KC290933** | 96.00 | 97.20 | 95.60 | 94.40 | 94.40 |  | **0.00** | 0.02 | 0.18 | **0.00** | 0.01 | 0.02 | 0.01 | 0.01 | 0.01 | 0.02 | 0.02 | 0.02 | **0.00** | 0.02 | 0.01 | 0.02 | 0.04 | 0.06 | 0.05 | 0.04 | 0.15 | 0.01 | 0.02 | 0.02 | 0.01 |
| **H2.2012.JX310862** | 96.40 | 97.60 | 96.00 | 94.80 | 94.80 | 99.60 |  | 0.02 | 0.17 | 0.01 | **0.00** | 0.02 | 0.01 | 0.01 | 0.02 | 0.01 | 0.02 | 0.02 | **0.00** | 0.02 | 0.02 | 0.02 | 0.03 | 0.05 | 0.05 | 0.04 | 0.14 | **0.00** | 0.02 | 0.02 | 0.01 |
| **H3.2012.KC290932** | 95.20 | 96.40 | 94.80 | 96.73 | 96.73 | 97.60 | 98.00 |  | 0.16 | 0.02 | 0.02 | **0.00** | 0.03 | 0.03 | 0.04 | 0.03 | 0.01 | 0.01 | 0.02 | 0.02 | 0.04 | **0.00** | 0.02 | 0.03 | 0.02 | 0.03 | 0.15 | 0.02 | **0.00** | 0.01 | 0.02 |
| **H4.2012.KC466019** | 81.60 | 82.80 | 81.60 | 85.12 | 85.54 | 84.00 | 84.40 | 85.37 |  | 0.18 | 0.17 | 0.16 | 0.19 | 0.19 | 0.20 | 0.19 | 0.16 | 0.17 | 0.17 | 0.19 | 0.20 | 0.17 | 0.19 | 0.17 | 0.15 | 0.20 | 0.35 | 0.17 | 0.16 | 0.18 | 0.18 |
| **M1.2012.KC290942** | 96.00 | 97.20 | 95.60 | 94.40 | 94.40 | 99.60 | 99.20 | 97.60 | 84.00 |  | 0.01 | 0.02 | **0.00** | **0.00** | 0.02 | 0.01 | 0.02 | 0.02 | 0.01 | 0.02 | 0.02 | 0.02 | 0.04 | 0.06 | 0.05 | 0.04 | 0.15 | 0.01 | 0.02 | 0.02 | 0.01 |
| **M2.2012.KC290934** | 96.40 | 97.60 | 96.00 | 95.18 | 95.18 | 99.20 | 99.60 | 98.39 | 84.74 | 99.20 |  | 0.01 | 0.01 | 0.01 | 0.02 | 0.01 | 0.02 | 0.02 | **0.00** | 0.01 | 0.02 | 0.01 | 0.03 | 0.05 | 0.04 | 0.03 | 0.14 | **0.00** | 0.01 | 0.01 | 0.01 |
| **M3.2012.KC290938** | 95.20 | 96.40 | 94.80 | 96.34 | 96.34 | 98.00 | 98.40 | 99.59 | 85.77 | 98.00 | 98.80 |  | 0.02 | 0.02 | 0.03 | 0.02 | **0.00** | 0.01 | 0.02 | 0.02 | 0.03 | 0.01 | 0.02 | 0.04 | 0.03 | 0.04 | 0.15 | 0.01 | **0.00** | 0.02 | 0.02 |
| **M4.2012.KC290941** | 95.60 | 96.80 | 95.20 | 94.00 | 96.71 | 99.20 | 98.80 | 97.20 | 83.60 | 99.60 | 98.80 | 97.60 |  | 0.01 | 0.02 | 0.02 | 0.03 | 0.02 | 0.01 | 0.02 | 0.02 | 0.02 | 0.04 | 0.06 | 0.05 | 0.05 | 0.15 | 0.01 | 0.02 | 0.02 | 0.01 |
| **M5.2012.KC290940** | 95.60 | 96.80 | 95.20 | 94.40 | 94.40 | 99.20 | 98.80 | 97.20 | 83.60 | 99.60 | 90.80 | 97.60 | 99.20 |  | 0.02 | 0.01 | 0.03 | 0.02 | 0.01 | 0.02 | 0.02 | 0.02 | 0.04 | 0.06 | 0.05 | 0.05 | 0.15 | 0.01 | 0.02 | 0.02 | 0.01 |
| **M6.2012.KC290935** | 94.80 | 96.00 | 94.40 | 94.00 | 94.00 | 98.80 | 98.40 | 96.40 | 82.80 | 98.40 | 98.00 | 96.80 | 98.00 | 98.40 |  | 0.01 | 0.04 | 0.03 | 0.02 | 0.03 | **0.00** | 0.03 | 0.05 | 0.07 | 0.06 | 0.05 | 0.15 | 0.02 | 0.03 | 0.03 | 0.02 |
| **M7.2012.KC290936** | 95.60 | 96.80 | 95.20 | 94.80 | 94.80 | 98.40 | 98.80 | 97.20 | 83.60 | 98.80 | 98.80 | 97.60 | 98.40 | 98.80 | 98.80 |  | 0.03 | 0.03 | 0.01 | 0.02 | 0.01 | 0.02 | 0.04 | 0.06 | 0.05 | 0.05 | 0.14 | 0.01 | 0.02 | 0.02 | 0.01 |
| **M8.2012.KC290937** | 94.80 | 96.00 | 94.40 | 95.93 | 95.93 | 97.60 | 98.00 | 99.19 | 85.37 | 97.60 | 98.39 | 99.59 | 97.20 | 97.20 | 96.40 | 97.20 |  | 0.01 | 0.02 | 0.03 | 0.04 | 0.01 | 0.03 | 0.04 | 0.03 | 0.04 | 0.16 | 0.02 | **0.00** | 0.01 | 0.02 |
| **M9.2012.KC290939** | 94.40 | 95.60 | 94.00 | 95.53 | 95.53 | 98.00 | 97.60 | 98.78 | 84.96 | 98.00 | 97.99 | 99.19 | 97.60 | 97.60 | 96.80 | 96.80 | 98.78 |  | 0.02 | 0.03 | 0.03 | 0.02 | 0.03 | 0.05 | 0.04 | 0.05 | 0.16 | 0.02 | 0.01 | 0.02 | 0.03 |
| **E.Mugla.2011.1*** | 96.40 | 97.60 | 96.00 | 94.80 | 94.80 | 99.60 | **100.0** | 98.00 | 84.40 | 99.20 | 99.60 | 98.40 | 98.80 | 98.80 | 98.40 | 98.80 | 98.00 | 97.60 |  | 0.02 | 0.02 | 0.01 | 0.03 | 0.05 | 0.05 | 0.04 | 0.14 | **0.00** | 0.02 | 0.02 | 0.01 |
| **E.Mersin.2011.1*** | 95.60 | 96.80 | 95.20 | 94.76 | 94.76 | 98.00 | 98.40 | 97.98 | 83.53 | 98.00 | 98.80 | 97.59 | 97.60 | 97.60 | 96.80 | 97.60 | 97.19 | 96.79 | 98.40 |  | 0.03 | 0.02 | 0.02 | 0.04 | 0.04 | 0.02 | 0.12 | 0.01 | 0.02 | 0.02 | 0.02 |
| **E.Mugla.2012.4*** | 94.80 | 96.00 | 94.40 | 94.00 | 94.00 | 98.80 | 98.40 | 96.40 | 82.80 | 98.40 | 98.00 | 96.80 | 98.00 | 98.40 | **100.0** | 98.80 | 96.40 | 96.80 | 98.40 | 96.80 |  | 0.03 | 0.05 | 0.07 | 0.06 | 0.05 | 0.15 | 0.02 | 0.03 | 0.03 | 0.02 |
| **E.Mugla.2011.9*** | 95.60 | 96.80 | 95.20 | 96.34 | 96.34 | 98.00 | 98.40 | 99.59 | 85.02 | 98.00 | 98.80 | 99.19 | 97.60 | 97.60 | 96.80 | 97.60 | 98.79 | 98.38 | 98.40 | 98.39 | 96.80 |  | 0.02 | 0.04 | 0.03 | 0.03 | 0.14 | 0.01 | 0.01 | 0.01 | 0.02 |
| **E.Adana.2011.1*** | 94.00 | 95.20 | 93.60 | 94.72 | 94.72 | 96.40 | 96.80 | 97.97 | 83.40 | 96.40 | 97.19 | 97.57 | 96.00 | 96.00 | 95.20 | 96.00 | 97.17 | 96.76 | 96.80 | 98.37 | 95.20 | 98.37 |  | 0.03 | 0.04 | 0.02 | 0.13 | 0.03 | 0.02 | 0.02 | 0.04 |
| **E.Adana.2011.2*** | 92.00 | 93.20 | 91.60 | 96.68 | 96.68 | 94.40 | 94.80 | 96.73 | 85.12 | 94.40 | 95.18 | 96.34 | 94.00 | 94.00 | 93.20 | 94.00 | 95.93 | 95.53 | 94.80 | 96.34 | 93.20 | 96.34 | 97.12 |  | 0.02 | 0.03 | 0.15 | 0.05 | 0.04 | 0.05 | 0.06 |
| **E.Mugla.2011.4*** | 92.80 | 94.00 | 92.40 | 95.71 | 95.71 | 95.20 | 95.60 | 97.55 | 86.72 | 95.20 | 95.98 | 97.15 | 94.80 | 94.80 | 94.00 | 94.80 | 96.75 | 96.34 | 95.60 | 96.36 | 94.00 | 97.15 | 96.33 | 98.33 |  | 0.05 | 0.17 | 0.04 | 0.03 | 0.04 | 0.05 |
| **E.Mugla.2011.2*** | 93.60 | 94.80 | 93.20 | 93.52 | 93.52 | 96.00 | 96.40 | 96.76 | 82.26 | 96.00 | 96.79 | 96.37 | 95.60 | 95.60 | 94.80 | 95.60 | 95.97 | 95.56 | 96.40 | 97.97 | 94.80 | 97.17 | 97.95 | 96.71 | 95.12 |  | 0.12 | 0.03 | 0.04 | 0.03 | 0.04 |
| **E.Mugla.2012.1*** | 84.40 | 85.60 | 84.00 | 84.68 | 84.68 | 86.80 | 87.20 | 86.69 | 72.29 | 86.80 | 87.55 | 86.35 | 86.40 | 86.80 | 86.80 | 87.20 | 85.94 | 85.54 | 87.20 | 88.62 | 86.60 | 87.10 | 87.76 | 86.48 | 85.02 | 89.67 |  | 0.14 | 0.15 | 0.14 | 0.15 |
| **M.Mersin.2011.1*** | 96.40 | 97.60 | 96.00 | 95.18 | 95.18 | 99.20 | 99.60 | 98.39 | 84.74 | 99.20 | **100.0** | 98.80 | 98.80 | 98.80 | 98.00 | 98.80 | 98.39 | 97.99 | 99.60 | 98.80 | 98.00 | 98.80 | 97.19 | 95.18 | 95.98 | 96.79 | 87.55 |  | 0.01 | 0.01 | 0.01 |
| **M.Mersin.2011.2*** | 95.20 | 96.40 | 94.80 | 96.34 | 96.34 | 98.00 | 98.40 | 99.59 | 85.77 | 98.00 | 98.80 | **100.0** | 97.60 | 97.60 | 96.80 | 97.60 | 99.59 | 99.19 | 98.40 | 97.59 | 96.80 | 99.19 | 97.57 | 96.34 | 97.15 | 96.37 | 86.35 | 98.80 |  | 0.02 | 0.02 |
| **M.Mersin.2011.3*** | 95.60 | 96.80 | 95.20 | 95.55 | 95.55 | 98.00 | 98.40 | 98.79 | 84.27 | 98.00 | 98.80 | 98.39 | 97.60 | 97.60 | 96.80 | 97.60 | 98.79 | 97.58 | 98.40 | 98.39 | 96.80 | 99.19 | 97.57 | 95.55 | 96.36 | 97.17 | 87.10 | 98.80 | 98.39 |  | 0.02 |
| **M.Edirne.2013*** | 96.00 | 97.20 | 95.60 | 94.40 | 94.40 | 98.80 | 99.20 | 97.60 | 84.00 | 99.20 | 99.20 | 98.00 | 98.80 | 98.80 | 97.60 | 98.80 | 97.60 | 97.20 | 99.20 | 98.00 | 97.60 | 98.00 | 96.40 | 94.40 | 95.20 | 96.00 | 86.80 | 99.20 | 98.00 | 98.00 |  |

(*: sequences characterized in this study. H: human sequences, E: equine sequences, M: mosquito sequences)
